# ^18^F-Glutathione Conjugate as a PET Tracer for Imaging Tumors that Overexpress L-PGDS Enzyme

**DOI:** 10.1371/journal.pone.0104118

**Published:** 2014-08-11

**Authors:** Ho-Lien Huang, Ying-Cheng Huang, Wei-Yuan Lee, Chun-Nan Yeh, Kun-Ju Lin, Chung-Shan Yu

**Affiliations:** 1 Department of Biomedical Engineering and Environmental Sciences, National Tsinghua University, Hsinchu, Taiwan; 2 Department of Neurosurgery, Chang-Gung memorial Hospital at Linkou, Chang Gung University, Taoyuan, Taiwan; 3 Department of Surgery, Chang-Gung memorial Hospital at Linkou, Chang Gung University, Taoyuan, Taiwan; 4 Department of Nuclear Medicine, Chang Gung Memorial Hospital at Linkou, Chang Gung University, Taoyuan, Taiwan; 5 Institute of Nuclear Engineering and Sciences, National Tsinghua University, Hsinchu, Taiwan; Chiba University Center for Forensic Mental Health, Japan

## Abstract

Lipocalin-type prostaglandin D synthase (L-PGDS) has been correlated with the progression of neurological disorders. The present study aimed at evaluating the imaging potency of a glutathione conjugate of fluorine-18-labeled fluorobutyl ethacrynic amide ([^18^F]FBuEA-GS) for brain tumors. Preparation of [^18^F]FBuEA-GS has been modified from the -4-tosylate derivative via radiofluorination in 5% radiochemical yield. The mixture of nonradioactive FBuEA-GS derived from a parallel preparation has be resolved to two isomers in a ratio of 9∶1 using analytic chiral reversed phase high performance liquid chromatography (RP-HPLC). The two fluorine-18-labeled isomers purified through nonchiral semipreparative RP-HPLC as a mixture were studied by assessing the binding affinity toward L-PGDS through a gel filtration HPLC, by analyzing radiotracer accumulation in C6 glioma cells, and by evaluating the imaging of radiotracer in a C6 glioma rat with positron emission tomography. The inhibition percentage of the production of PGD_2_ from PGH_2_ at the presence of 200 µM of FBuEA-GS and 4-Dibenzo[a,d]cyclohepten-5-ylidene-1-[4-(2H-tetrazol-5-yl)butyl]piperidine (AT-56) were 74.1±4.8% and 97.6±16.0%, respectively. [^18^F]FBuEA-GS bound L-PGDS (16.3–21.7%) but not the isoform, microsomal prostaglandin E synthase 1. No binding to GST-alpha and GST-pi was observed. The binding strength between [^18^F]FBuEA-GS and L-PGDS has been evaluated using analytic gel filtration HPLC at the presence of various concentrations of the cold competitor FBuEA-GS. The contrasted images indicated that the radiotracer accumulation in tumor lesions is probably related to the overexpression of L-PGDS.

## Introduction

According to Central Brain Tumor Registry of United States, brain tumor has emerged as the second and fifth to leading cause to death of adult male and of adult female, respectively, aging from 20 to 39 [1]. Whereas magnetic resonance imaging (MRI) is a useful clinical setting for noninvasive grading of brain tumor [2], evaluation of the treatment effects in malignant brain tumors is challenging [3]. Detection of those areas where the tumor progresses into the neighboring tissue highly depends on the extent and activity of this proliferation zone. These regions cannot often be distinguished from edema or necrosis by morphologic imaging modalities such as CT or MRI, however. Fluorine-18-labeled compounds have been used in imaging to detect tumors [4]–[6] and brain diseases [7]–[9]. The positron emitter ^18^F with its adequate half-life (t_1/2_) of 110 min emits two gamma photons at 180° generating images via positron emission tomography (PET) [10]. The fluorine-18-labeled compound, 2′-[^18^F]fluoro-2′-deoxy glucose {[^18^F]FDG}, has been widely used in imaging of tumors because of their high demand of glucose as the energy input to sustain the metabolism [11]. However, due to the higher background level in the normal brain region and non-biomarker-driven uptake features, efforts have been directed toward the development of thymidine kinase-targeted tracer, 3′-deoxy-3′-[^18^F]fluorothymidine {[^18^F]FLT} [12]. Although the accumulation level of [^18^F]FLT in the brain is relatively low, its higher tumor accumulation features make it a potential agent for diagnosis. Recent study of PET showed that a larger tumor volume could be detected using both [^18^F]FLT and [^11^C]methionine than that using gadolinium-enhanced MRI because increased transport and phosphorylation were always accompanied with the brain blood barrier disruption.

Due to the complexity associated with the tumor heterogeneity, search for a potential biomarker is of value. For example, the reactive oxidation species (ROS) are associated with the tumor progression [13]. One of the cellular defense mechanism is raised by releasing more glutathione (GSH), a tripeptide composed of cysteine, glutamic acid, and glycine. Up to date, regulation of this relatively concentrated antioxidant (ca. 2–3 mM in brain) has not been fully elucidated [14]. The downstream enzyme for GSH metabolism, e.g. glutathione transferase (GST) incorporates electrophilic substance to GSH for detoxification. Interestingly, catalysis for the conjugation of GSH is merely one of the three functions of GST. GSTs are composed of three subfamilies including cytosolic and mitochondrial GSTs as well as membrane associated proteins in prostaglandin and eicosanoid metabolism (MAPEG) [15]–[17]. Microsomal prostaglandin E synthase-1 (mPGES-1) is such as a member of MAPEG. The major role of cytosolic GST, which is the primary GST, is to detoxify the cell as described above. In addition to cytochrome P450 [18], cytosolic GST plays a housekeeping role in the liver.

GSH is not only utilized by GST enzymes, but it also functions as a cofactor for mPGES-1, an enzyme responsible for conversion of cyclooxygenase (COX)-derived prostanoid to prostaglandin E_2_ (PGE_2_) [19]–[21]. Analogous to mPGES-1 function, the lipocalin-type prostaglandin D synthase (L-PGDS) is expressed in the brain and is reportedly implicated in neurological disorders [22]. In addition, L-PGDS constitutes one of the most abundant proteins in the cerebrospinal fluid [23]. Unlike mPGES-1 which restricts recognition to only GSH analogs, the cofactors for L-PGDS, such as thio-containing molecules are relatively well tolerated. We thought that the conjugate of GSH e.g. [^18^F]FBuEA-GS **3** would be an adequate candidate compound for studying these enzymes. Inhibitor of COX has been associated with adverse side effects such as the gastric toxicity and the more seldom cardiovascular complications of prostacyclin loss. Targeting individual synthases downstream of COX such as L-PGDS represents a strategy to avoid these complications [24]. L-PGDS also binds retinoic acid, retinal, biliverdin, bilirubin, gangliosides and amyloid β peptides with high affinities of K_d_ = 20–200 nm, indicating that L-PGDS may act as a transporter protein of these lipophilic compounds and as an endogenous chaperon to prevent amyloid β aggregation [25]. 4-Dibenzo[a,d]cyclohepten-5-ylidene-1-[4-(2H-tetrazol-5-yl)butyl]piperidine (AT-56) is an orally active selective inhibitor of L-PGDS. AT-56 is competitive against PGH_2_ for catalysis by L-PGDS (K_M_ = 14 µM) with a K_I_ value of 75 µM in enzymatic assays [21]. AT-56 does not affect the activities of hematopoietic prostaglandin synthase (H-PGDS), COX-1, COX-2, or m-PGES-1 at concentrations up to 250 µM. However, such a functional assay may not virtually reflect the inherent binding affinities. Hence, [^18^F]FBuEA-GS **3** could be used as an innate probe to precisely analyze the binding patterns among these COX enzyme streams.

**Figure 1:**
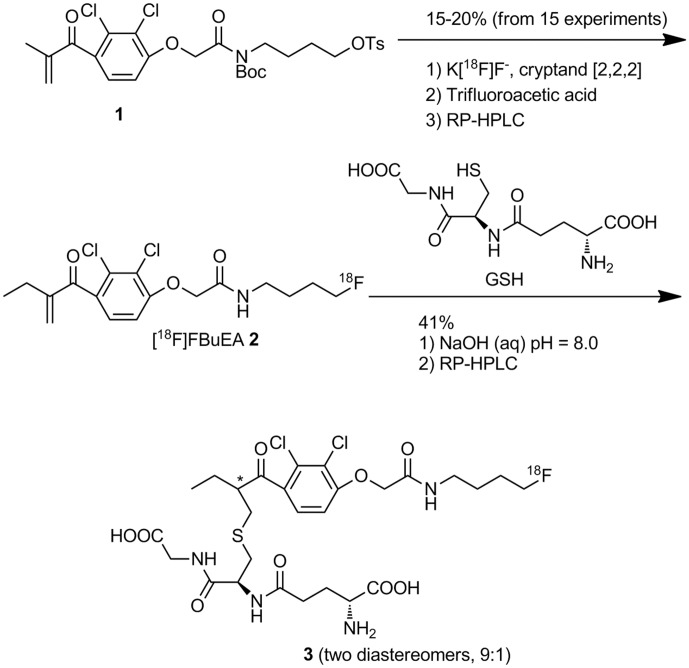
Preparation of [^18^F]FBuEA-GS 3 from tosylate 1 via a sequential two-step radiochemical synthesis followed by HPLC purification.

The introduction of the radioactive fluorine atom into potential biomolecules can be accomplished via nucleophilic [26]–[28] or electrophilic pathways [29]–[31]. Instead of calculating of the chemical yield based on the precursor, the radiochemical yield is based on the initial radioactivity of radiofluoride ^18^F^−^ (t_1/2_ = 110 min). The most common radiofluorine source, H[^18^F]F, is generated by bombarding the accelerated beam lines of protons on nonradioactive ^18^O-encapsulated H_2_O. Therefore, the synthetic design of a desired radiofluorinated compound aims to improve the radiochemical yield. Radioactive fluoride is commonly introduced using the S_N_2 reaction. The basicity of fluoride ions necessitates the protection of the remaining proton-containing acidic groups prior to the fluorination. Such a restriction is often encountered during the radiofluorination of amino acids and short peptides. Fluorine-18-labeled synthons [32]–[35] provide alternative routes to conjugated short peptides. Recently, we reported a method for preparing [^18^F]FBuEA **2** [36], a compound initially synthesized to study glutathione transferase (GST) activity, which may be upregulated in some tumors (Fig. 1) [37]–[40]. Hence, radiofluorination on the precursor to [^18^F]FBuEA **2** proceeding to the conjugation with the peptide GSH moieties could prevent from low radiochemical yield caused by the messy protection and deprotection procedures associated with the peptide moieties.

Interestingly, GSH has often been used as a model molecule to validate the conjugating ability of [^18^F]synthon [41]–[43]. However, the *in vivo* study of these conjugates has not been reported. Furthermore, because of its easy conjugation to GSH, we thought that [^18^F]FBuEA **2** could be introduced into other peptides that incorporate cysteine groups. Herein, we studied [^18^F]FBuEA-GS **3** using enzymatic binding and cellular uptake assays, *in vivo* radioactivity distribution, and *in vivo* PET imaging.

## Materials and Methods

### General

[^18^F]HF was produced with a PET tracer cyclotron (GE, TR-30) via the ^18^O(p,n)^18^F nuclear reaction at Nuclear Energy Research institute (NERI), Taiwan. The radiochemical experiment was performed with a GE TracerLAB FX_FN_ synthesis module (GE medical systems, Milwaukee, WI). The crude mixture [^18^F]FBuEA **2** in TracerLAB FX_FN_ synthesis module was purified using reversed phase high performance liquid chromatography (RP-HPLC), consisting of a Waters 510 pump and a linear UVIS detector (λ = 254 nm) in series with a Berthold γ-flow detector (Raytest, GABI Star) and a RP-18 column CHEMCOSORB 7-ODS-H, 10×250 mm, 5 µm. The identity of the labeled compound [^18^F]FBuEA **2** was confirmed by comparing with the authentic compound on HPLC chromatogram. The UV absorbance peak at 254 nm was integrated for comparing with the standard curve relating mass to UV absorbance. Only a specific activity below 40 GBq/µmol can be measured accurately. Radioactivity was measured with a Capintec R15C dose calibrator. Recombinant human glutathione S-transferase alpha-1 (GSTA1 human, 50 µg/50 µL) was purchased from Pro Spec-Tany Techno Gene Ltd (ENZ-469). Recombinant human glutathione S-transferase Pi-1 (GSTP1 human, 25 µg/25 µL) were purchased from Alpha Diagnostic International Inc. (GST P35-R-25). The enzymes of L-PGDS and m-PGES 1 were purchased from Cayman Chemical Inc. All these enzyme products were freshly unpacked and immediately used for enzymatic assay.

HPLC system used for binding assay included a Waters 510 pump and a linear UVIS detector (λ = 254 nm) that was assembled in series with a Berthhold γ-flow detector on a TSKgel G3000 PW 7.5×300 (mm) with a particle size of 10 µm, which was purchased from Tosoh Bioscience LLC. Flow rate was setting at 1 mL/min.

PET imaging was performed with microPET R4 (Concorde Microsystems Inc.) and a NanoPET/CT (MEDISO Inc.) in Nuclear Energy Research Institute. Both the machines were manufactured by Siemens Medical Solutions, Knoxville, United States.

### Radiochemical synthesis of [^18^F]FBuEA-GS 3

Preparation of compound **3** has been modified. In brief, [^18^F]FBuEA **2** was prepared from [^18^F]F^−^ (824 mCi) with the tosylate **1** through purification with a series of cartridge settings in a synthetic module. The fluorination agent was obtained from 3.5 mg K_2_CO_3_, 0.5 mL H_2_O and cryptand [2,2,2] (15 mg)/CH_3_CN (1 mL). In addition, *t*-BuOH (0.4 mL) was used during the fluorination procedure. The mixture of compound **2** was further purified using HPLC settings as described above. The flow rate was 3 mL/min. The gradient settings starts from 20% CH_3_CN (aq.) obtained by mixing CH_3_CN and 0.05% trifluoro acetic acid, via 95% CH_3_CN solution at 10 min, to a final 100% CH_3_CN solvent at 20 min. *t_R_* = 14.8 min. The preparation along with purification with semipreparative RP-HPLC was accomplished within 1 h. A portion (7 mCi, 0.2 mL) drawn off from the collected fractions (3 mL, 82 mCi) was transferred to a round-bottomed flask (10 mL) followed by concentration under reduced pressure using membrane pump to obtain the residue. To the residue was added CH_3_CN (1 mL), H_2_O (1 mL), and GSH (20 mg), sequentially. A solution of aqueous NaOH (50 mM) was added until the pH was adjusted to 8.2 (0.6 mL, within 1 min). The stirring was allowed for 15 min. After filtration with 0.45 µM Nylon filter (Merck), the filtrate (2.6 mL) was purified using semipreparative RP-HPLC. The column setting was the same as that described for compound **2**. The gradient settings were the same as that described above Retention time (t*_R_*) of [^18^F]FBuEA-GS **3** was 14.6 min. The fractions collected (6 mL) were concentrated under reduced pressure using membrane pump for 10 min to provide [^18^F]FBuEA-GS **3** in 5% radiochemical yield (2.05 mCi) with specific activity of 33 GBq/µmol and radiochemical purity of 98%, based on the calculation of initial radiofluoride ion [^18^F]F^−^ (824 mCi). For each group of experiment, a volume of 0.01 mL was drawn from a concentration of 440 µCi/0.2 mL of the purified [^18^F]FBuEA-GS **3**. Synthesis and purification of [^18^F]FBuEA-GS **3** from [^18^F]FBuEA **2** was completed within 1 h. The whole preparation along with purification with semipreparative RP-HPLC starting from radiofluoride ^18^F^−^ was completed in 2 h. Nonradioactive FBuEA-GS **3** was prepared separately and analyzed by RP-HPLC as described above except that an analytic chiral column (Chiralcel OD-RH 0.46×15 cm, Daicel Chemical Industries, LTD.) was used instead. The gradient setting was the same as above described and the flow rate was 0.7 mL/min.

### Bioassay of competitive inhibition of FBuEA-GS 3 against the production of PGD2 from PGH2

This assay was performed according to the protocol described by the commercial kit (Cayman cat. No. 10006595). In brief, this method was divided to two parts (Table S1 and S2). Part one was regarding the assay of production of PGD_2_ from PGH_2_ under the catalysis of L-PGDS. The formation of PGD_2_ could be inhibited by AT-56, a dibenzocycloheptenyl tetrazolyl piperidine. To compare with the inhibition by FBuEA-GS **3**, uridine was employed as a negative control. Part two was regarding the determination of the concentration of PGD_2_ by enzyme immune assay (EIA) of the PGD_2_-conjugate as a competitor. The conjugate linked by acetylcholineesterase and PGD_2_ binds competitively to an immobilized antibody. After wash, the residual conjugate could catalyze the hydrolysis of acetylcholine and the released thiocholine replaces one thio group of 5,5′-dithio-bis-2-nitrobenzoic acid yielding a colored 5-thio-2-nitrobenzoic acid with absorbance of UV at λ_max_ of 412 nm. The intensity of absorbance is inversely proportional to the concentration of PGD_2_ derived from PGH_2_. Thus, the more intensive absorbance the detector senses, the more effective inhibition the substrate exerts. Before performing the assay, a calibration curve by plotting the activity detected vs. concentration of PGD_2_ as the competitor was constructed (Fig. S1). Throughout the whole assay for the three substrates, the percentages of activities ranging from 41.4%–61.2% were lying in a reliable linear detection between 26.8% (15000 pg/mL) and 76.9% (468.8 pg/mL).The inhibition percentage was calculated as [(Abs_initial_ – Abs_control_)−(Abs_inhibitor_ – Abs_control_)]/(Abs_initial_ – Abs_control_)×100%. Experiments were performed in duplicate.

### Assay of binding of radioligand to enzymes tested

The aforementioned [^18^F]FBuEA-GS **3** was diluted with distilled H_2_O (1 mL). An aliquot (20 µL) was drawn off to each of the eppendorfs of the enzyme solution as indicated in Table S3. The whole mixture was incubated at 25°C for 15 min followed by analysis using HPLC coupled with gel filtration column (TSKgel G3000PW 7.5×300, 10 µm, Tosoh Bioscience LLC). Distilled H_2_O was employed as the eluent. The flow rate was 1 mL/min.

### Determination of binding constant (K_d_) of [^18^F]FBuEA-GS 3 to L-PGDS

An amount of 250 µg/200 µL of the commercial L-PGDS (human recombinant, Cayman, No. 10006788) was mixed with tris-HCl buffer (50 µL, 100 mM, pH = 8.0) to provide the stock solution (250 µg/250 µL). An aliquot (10 µL) drawn from the stock was added to an eppendorf (200 µL). A solution of [^18^F]FBuEA-GS **3** in tris-HCl buffer solution (0.40 µCi/5 µL) was added. A carrier solution of nonradioactive FBuEA-GS **3** was prepared via a series of dilution from a stock to provide various samples in concentration of 4, 30, 80, 600, 1600 and 4800 µM. A volume of 5 µL for each sample was added to the above eppendorf to generate the final concentrion of 1, 7.5, 20, 150, 400 and 1200 µM. As a control, 5 µL of tris-HCl solution was used. The mixture was immediately (5 sec.) transferred to HPLC for binding analysis. The other assay group using HPLC for equilibrium of 10 minutes followed the same condition except that the quilibrium time was extended to 10 min.

### Cell culture for C6 glioma and fibroblasts

A rat glioma cell line, C6 (Narotzky and Bondareff 1974), was initially obtained from American Type Cell Collection (ATCC). The C6 glioma cells were cultured with Dulbecco's modified Eagles medium (DMEM) with 10% of fetal calf serum (Gibco) under 5% of CO_2_ at 37°C in a 96 well microtiterplate. The cells were subcultured when reaching 80–90% of confluency. Fibroblast cell line, 3T3, was provided from our collaborator, Dr. Ya-Hwei Wu, by purchasing from ATCC (American Type Cell Collection). The cells were maintained using the same culture condition as that described for C6 glioma cells.

### Study of the cellular uptake of [^18^F]FBuEA-GS 3

The freshly prepared [^18^F]FBuEA-GS **3** was diluted with medium (DMEM, 5% FBS) to a concentration of 10 µCi/50 mL in a centrifuge tube. When the cells were grown in microtiterplates for 24 h, the growth medium (500 µL) was replaced with a mixture of [^18^F]FBuEA-GS **3** in 500 µL followed by incubation at 37°C. The time point of addition of radio tracer was staggered such that every group could be harvested concurrently. At various times of 0.25, 0.5, 1.5, 3 and 5 h, the collection of the medium was progressing. During harvesting, the radioactive medium was collected from each of the wells, followed by rinsing with PBS 500 µL twice. The medium and rinses (1.5 mL) were combined for counting; the counts were treated as extracellular radioactivity. Subsequently, the cells were lysed with 0.25% trypsin-EDTA (30 µL) and the wells were rinsed with PBS twice. Both cells and rinses (1.5 mL) were combined for counting; the counts were treated as intracellular radioactivity. Radioactivity was measured using a scintillation gamma counter (Packard 5000, Packard Instrument Co. laboratory) and decay was corrected. Samples were performed triplicated at each time point for all uptake studies. The uptake ratio was calculated according to the following expression:
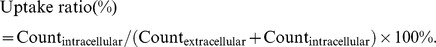



### Rat model

All *in vivo* experiments were performed in compliance with the NHMRC Taiwan Code of Practice for the care and use of animals for scientific purposes. Affidavit of approval of Animal Use Protocol Chang Gung Memorial Hospital, No 2013092702 and CGU12-055 was granted before performing the assessment. Sprague-Dawley (SD) rats (8 weeks of age) were obtained from the BioLasco animal Co. (Taiwan). Rats were housed under constant environmental conditions and were allowed free access to food and water throughout the experimental period. The rats were anaesthetized via inhalant isoflurane (Forthane, Abott) in 200 mL/min oxygen during the imaging study.

All studies involving animals were conducted in compliance with federal and institutional guidelines. Two weeks before imaging, healthy male SD rats were stereotactically inoculated in the right hemisphere with 1.0×10^5^ C6 glioma cells (American Type Tissue Collection). After C6 glioma cells were injected into the striatum of the SD rats, the animals were placed on heating pad until they have entirely recovered. When the xenografted tumor size has grown to a size of 1–2 mm in diameter, the animals were transferred to the animal facility under control by the research staff every morning. The animals were visited at least daily for signs of pain or distress; If the animals appear lethargic, do not appear to be eating or drinking over 24 hours, or weight loss greater than 20% body weight, euthanasia will be carried out to avoid further suffering. Prior to imaging, all rats were affixed with venous and arterial catheters.

The *in vivo* xenograft C6 glioma was imaged 2 weeks after transplantation procedure, until the volume of the tumor reached 3 mm–5 mm in diameter, well-demarcated from normal brain tissue.

Regular oral feeding was proceeded after the animals were recovered from anesthesia. The animals were monitored regularly with care in respect to the feeding quality, interaction, and symptom of dystrophy. The animal care unit controlled the abnormalities such as the feeding intake ratio less than 50% in 72 hours, hind leg paraparesis or the weight loss greater than 20%. As long as one of the above conditions was met, the animal will be sedated with the ketamine and xylazine hydrochloric acid combination followed by euthanasia with CO_2_ with xylocaine (200 mg) intravenously.

### *Ex vivo* analysis of the biodistribution of [^18^F]FBuEA-GS 3

Fourteen specimens were isolated after the injection of [^18^F]FBuEA-GS **3** in activity ranging from 0.9 to 1.2 mCi. The five rats were each used for provision of the specimens at the five time points of 15, 30, 60, 90 and 120 minutes post injection of [^18^F]FBuEA-GS **3**. The specimens included 1) organ tissues such as brain, liver, spleen, heart, kidney, lung, colon, small bowel, stomach, testes, skull, and muscle, and 2) body fluids such as blood and urine. These specimens were submitted for counting the radioactivity using a solid scintillation gamma counter (Packard 5000, Packard Instrument Co. laboratory). The counting value of each specimen was further divided by the sample weight to give the final expression as percentage of injection dose per sample weight (%ID/g).

### HPLC radiometabolite analysis

An amount of 2.14–2.72 mCi of [^18^F]FBuEA-GS **3** obtained as described above was dissolved in saline solution (0.2–0.3 mL). The injection dose for each of the 5 rats was in the range from 0.9 to 1.14 mCi per 0.1 mL except the group for 60 min experiment that used 0.3 mL. Arterial blood (2 mL) was collected at 15, 30, 60, 90, and 120 min from each of the 5 rats. After centrifugation with 3500 rpm at rt for 5 min, the supernatant (0.5 mL) was then mixed with the nonradioactive authentic FBuEA-GS (10 µL drawn from 1 mg/1 mL) followed by semipreparative RP-HPLC investigation using the gradient condition as described above. Radiochromatographic data were recorded and collected using a radioisotope detector (Bioscan, Washington, DC, USA).

### Immunohistological stainings

The whole brains of a rat were harvested and fixed in 4% formalin for 48 hours followed by paraffin embedding for immunohistological stainings. Tissue sections were detached in thickness of 5.0-µm followed by staining with the kit of L-PGDS-specific rabbit polyclonal antibody (Novus, NBP1-79280). Immunoactive spots were assessed using a horseradish peroxidase detection kit (Dako, Glostrup, DK). Hematoxylin and eosin staining was used to evaluate the cell density and tumor localization.

### MRI Imaging

MRI was used to localize the site of C6 tumor lesions. Rats were secured prone in a radiofrequency coil (38-mm inner diameter) and placed in a 4.7-T horizontal bore imaging system (Varian Inc., Palo Alto, CA, USA). A constant body temperature at 37°C was maintained using heated airflow. An initial multislice gradient-echo imaging sequence (repetition time, 150 ms; echo time, 3.5 ms; matrix, 128×128; field of view, 40×40 mm^2^; slice thickness, 2 mm) was used to acquire 7 slices for each of axial, coronal and sagittal imaging plane for proper positioning of subsequent scans. A multislice T2-weighted fast spin-echo scan with 8 echoes and 8.0-ms echo spacing (effective echo time, 32 ms) was then collected using the parameters of a repetition time of 2,000 ms, field of view of 32×32 mm^2^, matrix of 128×128, 16 acquisitions and 8 coronal slices in thickness of 2-mm.

### PET/CT Imaging

PET scanning experiments were performed within 72 hours of MRI experiment that used to confirm a successful inoculation of tumors by administering [^18^F] FBuEA-GS **3** *via* tail vein injection. Both machines of microPET and nanoPET/CT were employed. Data were collected in list-mode format for 120 minutes. For reconstruction, the dynamic PET acquisition was divided into six 20-min frames over the scanning duration. The raw data within each frame were then binned into three-dimensional sinograms, with a span of three and ring difference of 47.The data were corrected for scattering and attenuation using a two-dimensional ordered-subsets expectation-maximization algorithm with 16 subsets and four iterations. The sonograms were reconstructed into tomographic images (128×128×95) with voxel sizes of 0.095×0.095×0.08 cm^3^.

## Results and Discussion

### Radiochemical synthesis

**Figure 2:**
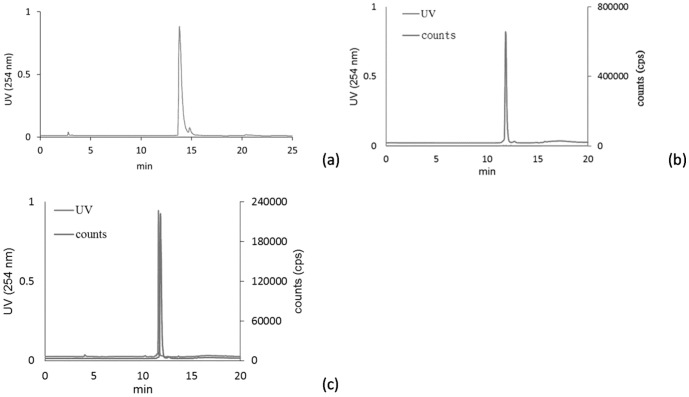
Purification using chiral analytic RP-HPLC and semipreparative RP-HPLC. (a) The racemic mixture of nonradioactive FBuEA-GS 3 was resolved to two components by chiral analytic RP-HPLC. The major peak A and the minor peak B represent the presence of two isomers. Injection volume: 0.01 mL from the sample with concentration of 1 mg/0.2 mL. (b) A typical chromatogram of [18F]FBuEA-GS 3 after purification with semipreparative RP-HPLC. Injection volume: 0.01 mL from the purified sample with concentration of 440 µCi/0.2 mL. (c) The HPLC chromatogram of the purified [^18^F]FBuEA-GS **3** co-mixed with the authentic sample using semipreparative RP-HPLC. Injection volume: 0.2 mL from authentic sample with concentration of 0.02 mg/0.2 mL.

Preparation of [^18^F]FBuEA **2**, the intermediate to [^18^F]FBuEA-GS **3**, has been modified. In general, [^18^F]FBuEA **2** was obtained in radiochemical yields ranging from 20 to 30%, resulting from a batch of more than 15 experiments. In contrast to the usual fluorinating agent [^18^F]Bu_4_NF (TBAF), combination of K[^18^F]F and cryptand [2,2,2] was used. Furthermore, we found that although t-BuOH did not improve the radiochemical yield, no failure was encountered during the fluorination. [^18^F]FBuEA-GS **3** could be easily formed via the conjugation of [^18^F]FBuEA **2** with GSH by merely adjusting pH = 8.0. Nonradioactive FBuEA-GS **3** obtained from a parallel experiment could be resolved into two isomers in a ratio of 9∶1 using analytic chiral HPLC (Fig. 2a). For preparation purposes, a mixture of the two isomers of [^18^F]FBuEA-GS **3** obtained from semipreparative RP-HPLC purification was promptly used for all experiments, including radioligand enzymatic binding assays, cellular uptake study, *ex vivo* biodistribution experiments, and *in vivo* PET studies. No further isolation of the two isomers with chiral RP-HPLC was resumed. From a series of experiments, [^18^F]FBuEA-GS **3** was obtained from [^18^F]F^−^ (end of bombardment, EOB), resulting in a radiochemical yield of 5%. Its specific activity and radiochemical purity were determined to be 33 GBq/µmol and 98% (Fig. 2b), respectively.

### Bioassay of the competitive inhibitor FBuEA-GS 3 against the production of PGD2

**Figure 3:**
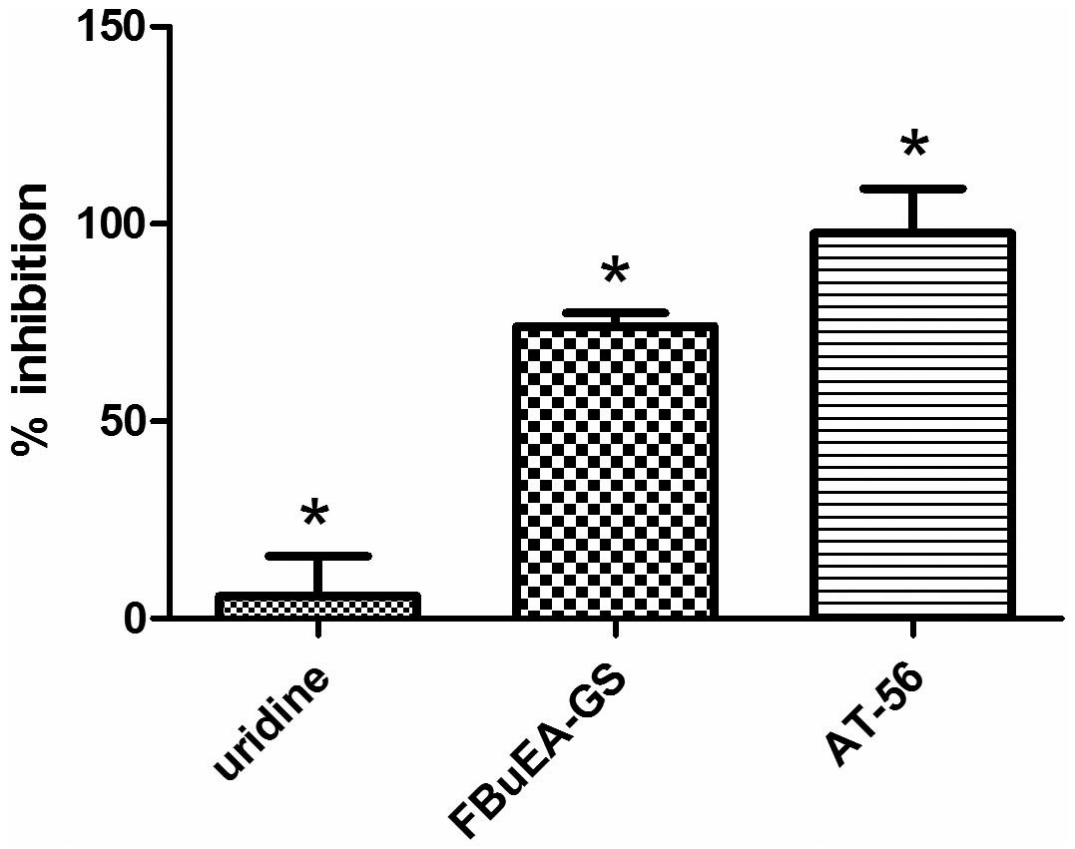
Inhibition of the formation of PGD_2_ from PGH_2_ in the presence of 200 µM of each test compound. Results are the mean of duplicated measurements.

To date, there is still no effective inhibitor of L-PGDS except AT-56 (IC_50_ = 95 µM) [25], a dibenzocycloheptenyl tetrazolyl piperidine. This assay was performed via an indirect determination of the formation of PGD_2_ in the presence of the competitive PGD_2_- acetylcholineesterase conjugate, which cleaves acetylthiocholine and the substrate 5,5′-dithiobis(2-nitrobenzoic acid) to yield a colored 5-thio-2-nitrobenzoic acid with an absorbance of visible light at a λ_max_ of 412 nm. According to the IC_50_ value of AT-56 [44], working concentrations of 200 µM of substrates were required to ensure that AT-56 could be used as a positive control (Fig. 3). The relatively large deviation of uridine (5.6±14.3%) reflects the complexity of sequential assays. The observed inhibition was relatively higher than that observed in previous studies. Compared to the AT-56 positive control that, showed complete inhibition (97.6±16.0%), FBuEA-GS **3** (74.1±4.8%) data were significant.

### Radioligand enzymatic binding assays

There were several rationales for selecting enzymes to be used in the radioligand enzymatic binding assays with [^18^F]FBuEA-GS **3**. L-PGDS catalyzes the oxidation of prostaglandin H_2_ (PGH_2_), a metabolite (also known as a prostanoid) derived from arachidonic acid (AA) through oxidation and reduction via the catalysis of COX enzymes. Because of the sequential catalysis of AA analogs upon receiving stimulus, L-PGDS and COX enzymes could exert similar binding affinities toward [^18^F]FBuEA-GS **3**. Hence, radioligand-binding experiments [45] also involved COX-1 and COX-2 enzymes. For comparison, mPGES-1, the counterpart of L-PGDS that catalyzes the formation of PGE_2_ from COX-derived PGH_2_, was also used in this study. GST enzymes catalyze the conjugation of GSH to [^18^F]FBuEA **2** without having any significant binding to its metabolite [^18^F]FBuEA-GS **3**. Thus, GST-P1 and GST-A1-1 enzymes were only used as negative controls to test our hypothesis.

To perform this assay, the concentrations of the enzymes could not be leveled off due to different commercial sources. The specific activities of the enzymes are in the following order: COX-1 (20 units/µL)>COX-2 (7.8 units/µL)>mPGES-1 (2.2 units/µL)>>L-PGDS of three species (2.4×10^−3^ units/µL)>GST-P1≈GST-A1-1 (5×10^−4^ units/µL).

**Figure 4:**
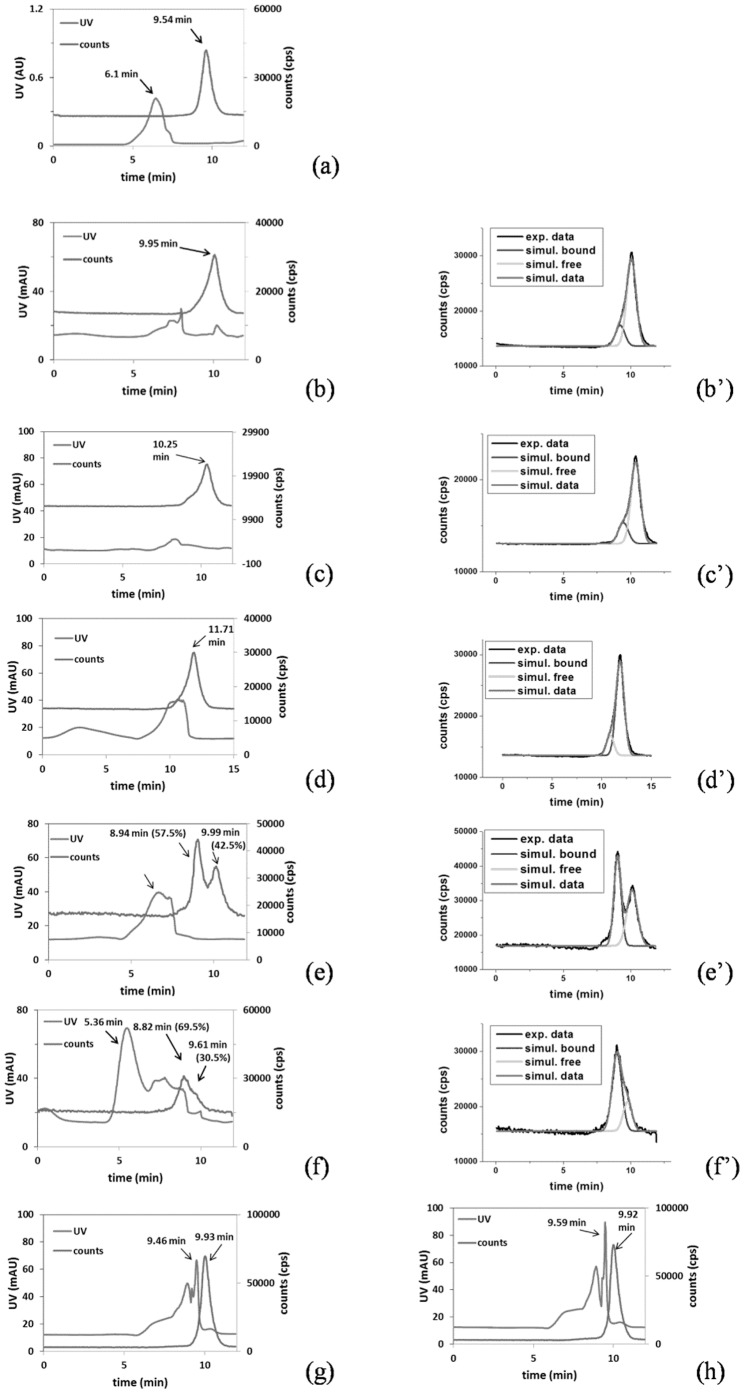
HPLC chromatograms for the mixture of [^18^F]FBuEA-GS 3 with different enzymes. (a) mPGES-1, (b) L-PGDS (lipocalin-type; rat recombinant), (c) PGDS (lipocalin-type; human recombinant), (d) PGDS (lipocalin-type; mouse recombinant), (e) COX-1 (ovine), (f) COX-2 (ovine), (g) GSTA1-1 and (h) GSTP1. (b′)∼(f′) are chromatograms resolved from the radioactivity signals of (b)∼(f) using Origin software.

Based on the results of the binding study (Fig. 4 and Table 1), COX enzymes tolerated the substrate with structural variation. The binding ratios of 52% and 75% for COX-1 and COX-2 enzymes, respectively, were significantly higher than those of the other enzymes. The specific activities of the three L-PGDS were significantly lower than those of the COX enzymes, and substantial binding was observed across all three species. Interestingly, mPGES-1 with a 1000-fold greater specific activity than that of L-PGDS did not show any binding affinity. The weak binding affinities of GSTA1-1 and GSTP1 could not be rationalized by lower specific activities because L-PGDS had similar specific activities (5-fold excess) and exhibited substantial binding. Thus, the binding sites of both mPGES-1 and GSTs may be restricted to GSH by the substrates, whereas COX and L-PGDS tolerate structural variances of substrates. Furthermore, L-PGDS recognized diversified thiol-containing structures as cofactors, enhancing the binding. The weak binding of both GSTs to [^18^F]FBuEA-GS **3** was consistent with our hypothesis.

**Table 1:** Tabulated binding affinities of enzymes to [^18^F]FBuEA-GS **3** determined from chromatograms of gel-filtration HPLC in Fig. 3.

enzyme	*COX -1*	COX -2	m-PGES	L-PGDS mouse recombinant	L-PGDS rat recombinant	L-PGDS human recombinant	GST-α	GST-π
**binding ratio (%)**	*52*	74.2	negligible	16.3	19.2	21.7	negligible	negligible

Binding ratios were calculated according to the peak integral of the green peak divided by the sum of the green and red peak integrals.

**Figure 5:**
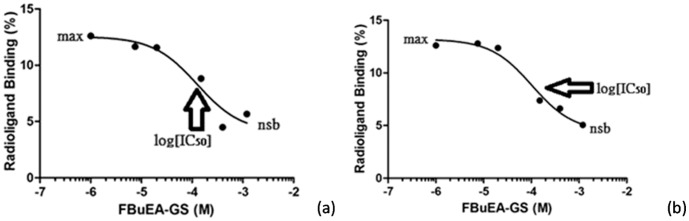
Inhibition curves of the binding of [^18^F]FBuEA-GS 3 to L-PGDS (human) at the presence of the inhibitor FBuEA-GS 3 in various concentrations. Data was obtained from single measurement. (a) The plot of 5 sec. equilibrium. The maximal binding ratio of the control group was 16% and the non-specific binding (nsb) ratio was 6%. (b) The plot of 10 min. equilibrium. The maximal binding ratio was 13% and the non-specific binding (nsb) ratio was 5%.

Because [^18^F]FBuEA-GS **3** binds significantly toward L-PGDS, we wish to use this HPLC analysis (Fig. S2, S4) to approach the binding constant K_d_ (Fig. 5). The concept of competitive inhibition using a radioactive ligand to study the dependence of the radioligand-receptor binding on the cold inhibitor is well established [46]. The inhibition curves were generated by plotting the radioligand binding ratio vs. concentration of FBuEA-GS **3** added. The software GraphPad Prism 5 with the nonlinear regression mode was used to generate the fittings. IC_50_ value was derived from the half way between the non-specific binding (nsb) asymptote and the maximum binding asymptote. Whereas the measurement was commonly taken for 15 min equilibrium, a very short equilibrium (5 sec.) was tested for comparison. The IC_50_ values for the two equilibrium experiments of 5 sec. mode and 10 min. mode were 120 µM and 101 µM, respectively. The IC_50_ values derived from very short mixing time (5 sec.) and the longer mixing time (10 min) are comparable implying a rapid equilibrium between the enzyme and substrate.

It has been reported that IC_50_ = [L]_cold_ = [L*]+K_d_. Hence, the present measurement employs 0.4 µCi of [^18^F]FBuEA-GS **3** i.e. 1.16×10^−18^ mole. From the derivation, the concentration of [L*] was 1.1×10^−5^ µM that was much less than the IC_50_ values of 120 and 101 µM as described above. Hence, K_d_ is equal to IC_50_. A high [L*] will bias the sensitivity to K_d_. The high specific activity of radiofluorine ensures a very low concentration of [^18^F]FBuEA-GS **3** used for the current assay. Thus, an acceptable counting statistics for accurately assaying separated [L*] and [L*R] from HPLC chromatogram was generated.

The relatively large K_d_ (110 µM) values was probably due to a new equilibrium in gel filtration column that has been reestablished during HPLC analysis. In contrast to the common binding experiment that employs infiltration method for single solid-liquid distribution for equilibrium [47], our analysis with column chromatography carried out a series of solid-liquid distribution, i.e. much more theoretic plates, facilitating the thermodynamic equilibrium. Thus, the K_d_ value derived from the typical binding assay is expected to be lower than the present HPLC binding experiment.

### Biological testing

**Figure 6:**
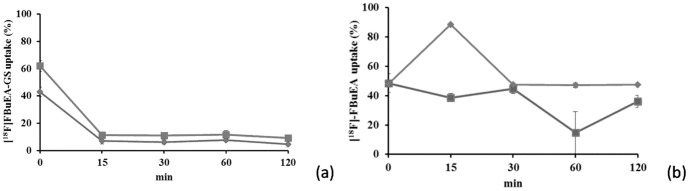
Comparison between the cellular uptake of two radiotracers. The radioactivity uptake of [^18^F]FBuEA-GS **3** (a) and [^18^F]FBuEA **2** (b) by C-6 tumor cells (redline) and fibroblasts (blue line).

**Figure 7:**
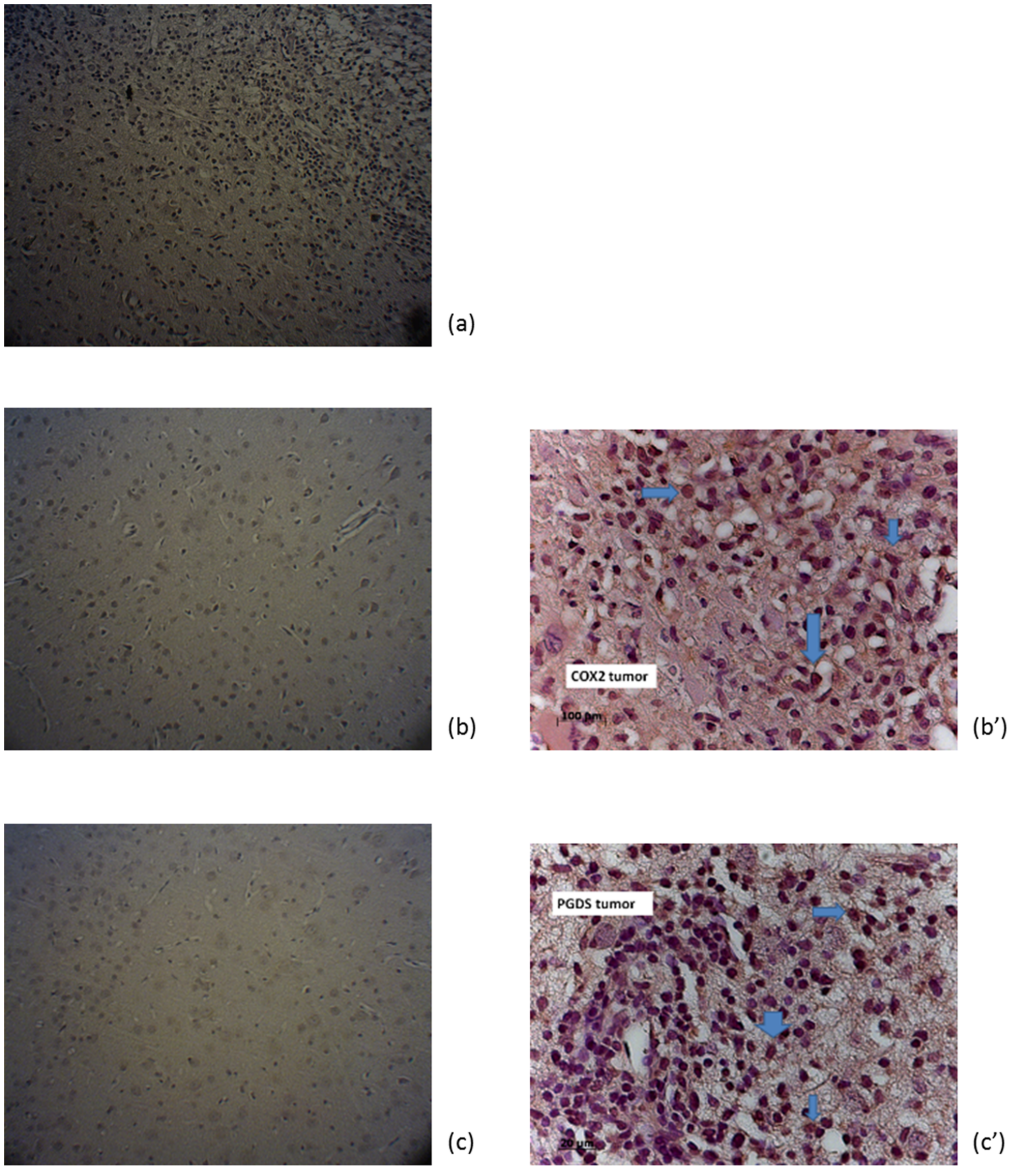
Immunohistological staining of COX enzymes and L-PGDS in both tumor and normal tissues. (a) COX1 staining- tumor, (b) COX2 staining- normal, (b′) COX2 staining- tumor, (c) L-PGDS staining- normal, (c′) L-PGDS staining- tumor.

As shown in Fig. 6a, the accumulation of radioactivity of [^18^F]FBuEA-GS **3** was higher in tumor cells compared to that of normal cells (9% vs. 6%). Although the difference in tracer uptake between C6 glioma and fibroblast lies within the statistic error (p<0.001 at 0 min and p>0.05 at rest time points), the accumulation level in C-6 glioma cell is higher. The accumulation pattern also differed from that of [^18^F]FBuEA **2**, which had a lower uptake in tumor cells compared to normal cell (Fig. 6b). These data indicate that the higher tumor cell uptake of [^18^F]FBuEA-GS **3** was due to the GSH moiety. However, the accumulation levels of radioactivity in tumor and normal cells decreased at late stages, which may imply that an initial supply of GSH was required by both cells in order for early antioxidation to maintain homeostatic functions. After reaching a steady state (approximately 15 min), the preferential radioactivity accumulation in tumors cell was maintained but then steadily decreased. The aforementioned insignificant difference in tracer uptake was also observed in that case of [^18^F]fluorothymidine ([^18^F]FLT); 4% vs. 3% for tracer uptake in the two cells (unpublished work). [^18^F]FLT is nevertheless a potential tracer for brain tumor imaging as described in the introduction part. The higher tracer accumulation in tumor cells at a later stage may imply an overexpression of GSH-binding membrane proteins. Thus, immunohistological staining for L-PGDS and COXs enzymes of tumor cells and normal cells was performed (Fig. 7). The results showed that, with the exception of COX-1, L-PGDS and COX-2 were both overexpressed in tumor cells.

**Figure 8:**
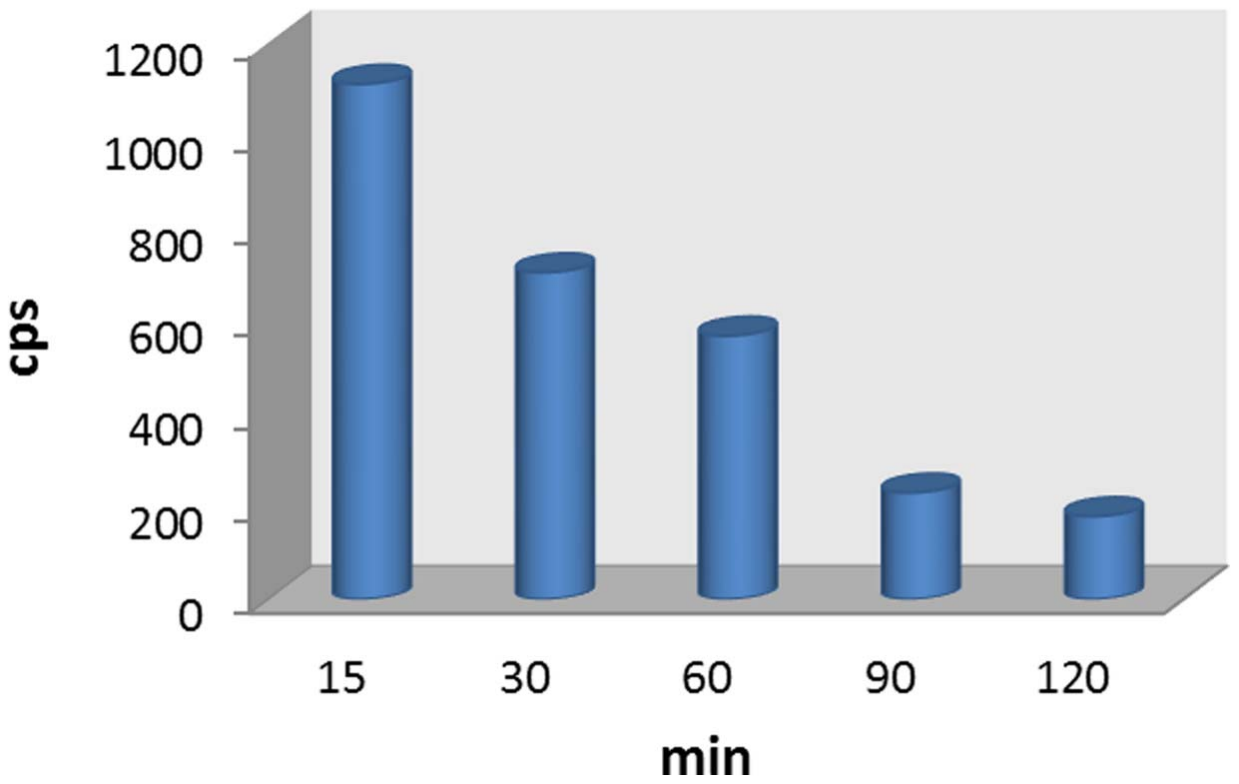
Bar diagram for the radiometabolite analysis of the radiotracer using semipreparative RP-HPLC. The blood samples were taken at various time points after [^18^F]FBuEA-GS **3** was injected intravenously.

The radiometabolite of [^18^F]FBuEA-GS **3** was analyzed by semipreparative RP-HPLC (Fig. S6). Blood samples were taken from each rat at various times post injection. The subsequent HPLC chromatogram of the sample showed an identifiable peak corresponding to [^18^F]FBuEA-GS **3**. The peaks of [^18^F]FBuEA-GS **3** in all chromatograms obtained from various time points were integrated and the radioactivity counts were plotted against time points (Fig. 8). The *in vivo* haliflife (t_1/2_) of [^18^F]FBuEA-GS **3** was determined to be 60 min.

Before performing *in vivo* assays, we summarized our findings from the above experiments. In Fig. 5a, the *in vitro* accumulation of radioactivity showed that the rapid decrease in accumulation may be due to perfusion effects leading to equilibrium. However, a closed *in vitro* system was unlikely to allow for such an efficient release and washout. If this was the case, the accumulation levels for both cell lines would have reached the same level. γ-GT enzymes recognize GSH analogs to enable efficient cleavage, which may have been observed in the *in vivo* radiometabolite analysis (*t_R_* = 23 min, Fig. S6) because a metabolite with a polarity between that of [^18^F]FBuEA-GS **3** and [^18^F]FBuEA **2** appeared at the chromatogram. Hence, a relatively higher uptake in tumor cells could be due to the overexpression of some enzymes. This was confirmed by the immunohistological staining results (Fig. 7).

**Figure 9:**
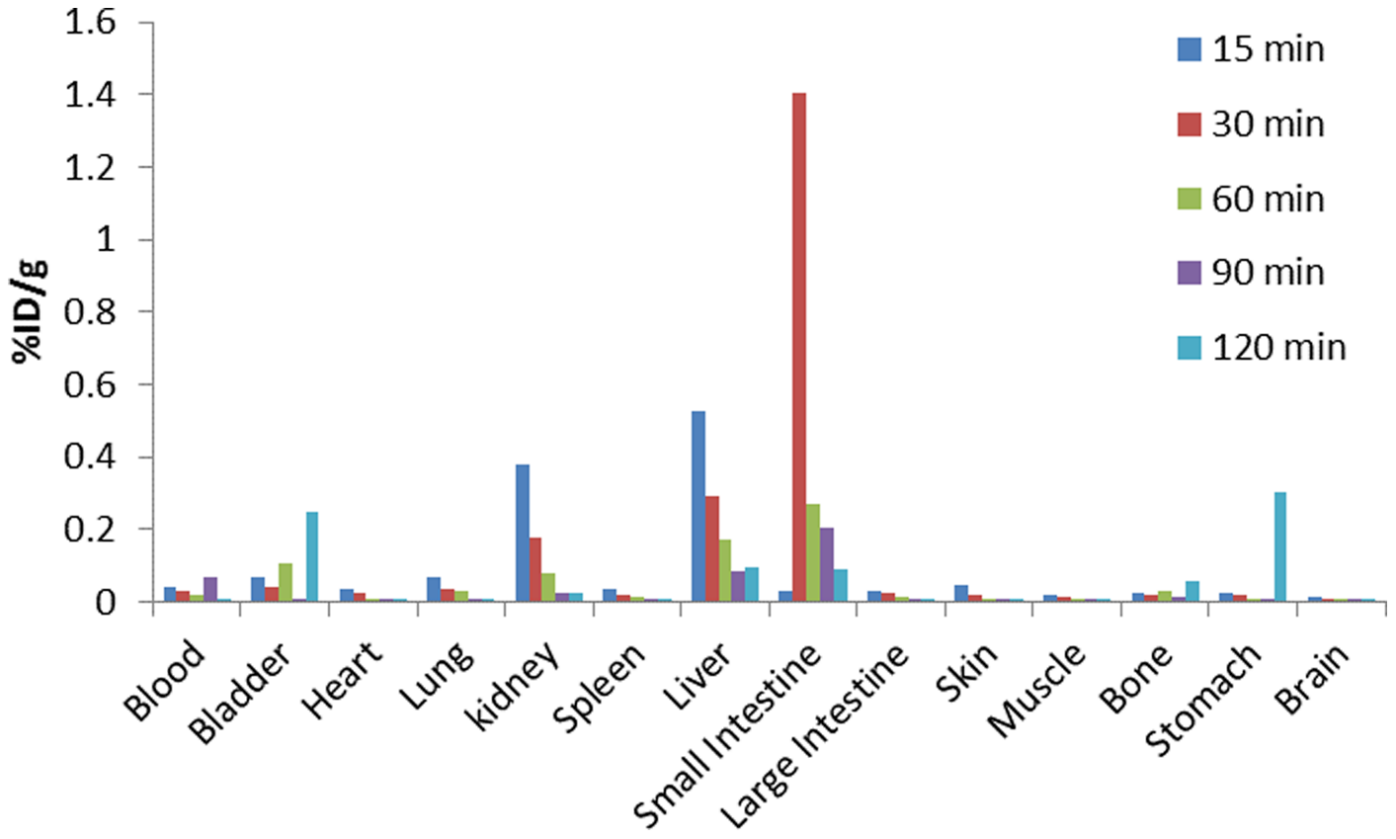
*Ex vivo* analysis of the distribution of [^18^F]FBuEA-GS 3 in a rat.

**Figure 10:**
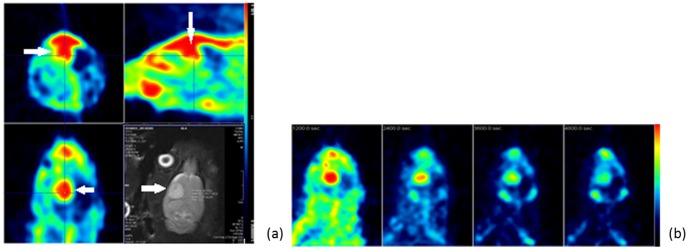
Images of PET and MRI of brain of a C6-glioma rat. (a) Dynamic PET images taken over 20–40 min at three cross sections. Lower right shows an MRI image. (b) Images of the coronal cross section at different time frames (10–30 min, 30–60 min, 60–90 min and 90–120 min) post injection of [^18^F]FBuEA-GS **3**. PET scanner description: microPET R4; Concorde Microsystems Inc. Injection dose: 1.58 mCi/0.5 mL.

Based on the half-life of 1 h, the *in vivo* PET imaging test using micro PET were used in a 2-hr dynamic study, and the distribution of radioactivity in a rat was determined (Fig. 9). Fourteen different tissue samples were collected for the biodistribution study of 1.0–1.5 mCi of [^18^F]FBuEA-GS **3** injected in a rat. The radioactivity was mainly localized in the excretory system. Only a limited amount of compound **3** was found in the brain (0.05%ID/g). Because of the quantitative features of PET, the radioactivity in tumor and normal tissues could be differentiated. [^18^F]FBuEA-GS **3** was subsequently evaluated as a tracer for imaging a rat with a brain tumor (Fig. 10 and Fig. S8). The tumor was successfully inoculated in the upper right part of the brain as confirmed by MRI imaging. The same rat was then taken for the measurement of gamma photons emitted from [^18^F]FBuEA-GS **3**, which was injected within 72 h after MRI imaging. The reconstructed images from all three cross sections showed a clear hot spot coinciding to the tumor region detected by MRI imaging. The dynamic PET images at coronal section from 0–120 min indicated that both the signal intensities on the tumor and regions other than tumor lesion decreased concomitantly. This may indicate a washout of [^18^F]FBuEA-GS **3**, resulting in a homeostatic function maintained by a rapid equilibrium. The imaging results were consistent with the results of the *in vitro* radioactivity accumulation study (Fig. 6a). An observable contrasted hot spot on the tumor lesion may indicate relatively weak binding by [^18^F]FBuEA-GS **3** or that limited expression of L-PGDS dominates the lateral equilibrium. The imaging experiments have been performed twice independently for two different C6-glioma rats using two PET scanner machines. Both the radioactivity accumulation levels in tumor lesions of the two rats are obvious but quantitative comparison has not been carried out. The difference between PET-scan images from the two types of PET scanners might be due to the differential threshold value set by micro PET and nano PET-CT (Fig. 10 and Fig. S8). Furthermore, the images of nano PET-CT were constructed using time frames from 0 to 60 min that might modify the signal intensity around the tumor lesion. In addition, the diminished radioactivity accumulation levels as shown in images of Fig. S8 may be due to the lower expression level of L-PGDS enzymes in this rat but not due to the lower efficiency of the PET scanner settings.

The hydrophilicity of [^18^F]FBuEA-GS **3** may limit its access to intracellular space. However, the nucleoside analog [^18^F]FLT with both higher polarity and lack of an adequate transporter is still intracellularly trapped via phosphorylation. There are a number of factors may override the drawbacks of these polar compounds for in vivo application e.g. the relatively lower background, the fast plasma clearance rate, the *in vivo* stability as well as formation of the more polar metabolite. At current stage, attention is drawn to study ex vivo analysis and the quantitative PET analysis. Both of the works are in due course.

## Conclusions

In brief, we have prepared [^18^F]FBuEA-GS **3** using an acceptable amount of radioactivity that can be used for *in vitro* and *in vivo* imaging studies. The multi-binding roles of [^18^F]FBuEA-GS **3** need to be further examined for future studies of the cyclooxygenase pathway-related disease models. Although the hot spot of the PET images may be due to the lower expression level of L-PGDS or the inferior binding, the binding affinity (K_d_ = 110 µM) using HPLC analysis that may be undervalued should not be overlooked. In addition, specific inhibitors and improved methods of radiochemical preparation need to be developed. The present findings suggest that [^18^F]FBuEA-GS **3** may be potentially used to image the expression of L-PGDS, which has been related to Parkinson's disease [48].

## Supporting Information

Figure S1 **Calibration curve of the activity detected vs. PGD2 as the standard at various concentration.**
(TIF)

Figure S2 **HPLC chromatogram of the binding analysis of [^18^F]FBuEA-GS 3 to L-PGDS.** Nonradioactive FBuEA-GS **3** of (a) 0 µM, (b) 1 µM, (c) 7.5 µM, (d) 20 µM, (e) 150 µM, (f) 400 µM and (g) 1200 µM were used for the version of 5-sec. equilibrium. AU = arbitrary unit; mAU = 10^−3^×arbiturary unit. (a′)∼(g′) are chromatograms resolved from the radioactivity signals of (a)∼(g) using Origin software.
(TIF)

Figure S3 **Illustration of the reponse of the UV absorption on the concentration of FBuEA-GS 3 in each HPLC chromatogram of Fig. S2.**
(TIF)

Figure S4 **HPLC chromatogram of the binding analysis of [^18^F]FBuEA-GS 3 to L-PGDS.** Nonradioactive FBuEA-GS **3** of (a) 0 µM, (b) 1 µM, (c) 7.5 µM, (d) 20 µM, (e) 150 µM, (f) 400 µM and (g) 1200 µM were used for the version of 10-min. equilibrium. AU = arbitrary unit; mAU = 10^−3^×arbiturary unit. (a′)∼(g′) are chromatograms resolved from the radioactivity signals of (a)∼(g) using Origin software.
(TIF)

Figure S5 **Illustration of the reponse of the UV absorption on the concentration of FBuEA-GS 3 of each HPLC chromatogram in Fig. S4.**
(TIF)

Figure S6 **RP-HPLC analysis of the radiometabolites from various blood samples at (a) 15 min, (b) 30 min, (c) 60 min, (d) 90 min and (e) 120 min post injection.**
(TIF)

Figure S7 **Immunohistological stainings for COXs and L-PGDS enzymes.** (a) COX-1 staining of normal brain tissue, (b) COX1 staining- tumor center, (c) COX2 staining- tumor/brain margin, (d) L-PGDS staining- tumor/brain-100×.
(TIF)

Figure S8 **Fused CT-PET images of a C6-glioma rat for confirmation of the tumor implantation using the second PET scanner (nanoPET/CT, MEDISO Inc).** From left to right: sagittal image, coronal image and transverse image. Injection dose: 1.085 mCi/0.2 mL. Images were taken from the mean of 0–60 min.
(TIF)

Table S1 **Protocols for formation of PGD2.**
(DOCX)

Table S2 **Protocols for enzymatic immunological assay.**
(DOCX)

Table S3 **Enzymes used for radioactive ligand binding assay.**
(DOCX)

Table S4 **Tabulation for the response of the UV absorption on the concentration of FBuEA-GS 3 of each HPLC chromatogram in Fig. S2.**
(DOCX)

Table S5 **Tabulation for the response of the UV absorption on the concentration of FBuEA-GS 3 of each HPLC chromatogram in Fig. S4.**
(DOCX)

Table S6 **Tabulation for the integrals of the peaks corresponding to the bounded fom and free form in the HPLC chromatogram of [^18^F]FBuEA-GS 3 with L-PGDS.**
(DOCX)
