# Supplementary figures and images for: 18F-Glutathione Conjugate as a PET Tracer for Imaging Tumors that Overexpress L-PGDS Enzyme
Source: PLoS One. 2014 Aug 11;9(8):e104118. doi: 10.1371/journal.pone.0104118 (PMC4128654; doi:10.1371/journal.pone.0104118)

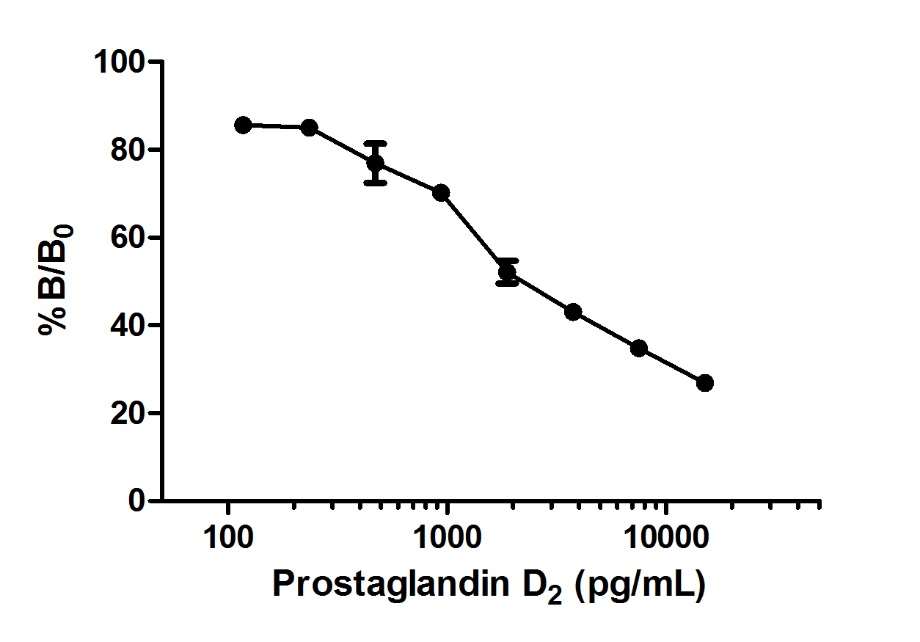

Supplement: Figure S1 — Calibration curve of the activity detected vs. PGD2 as the standard at various concentration. (TIF) [file pone.0104118.s001.tif]

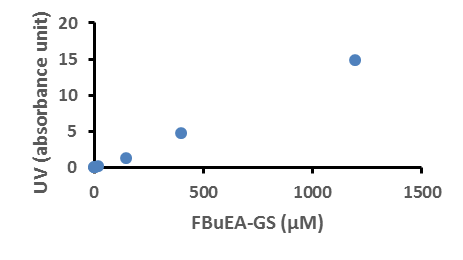

Supplement: Figure S3 — Illustration of the reponse of the UV absorption on the concentration of FBuEA-GS 3 in each HPLC chromatogram of Fig. S2. (TIF) [file pone.0104118.s003.tif]

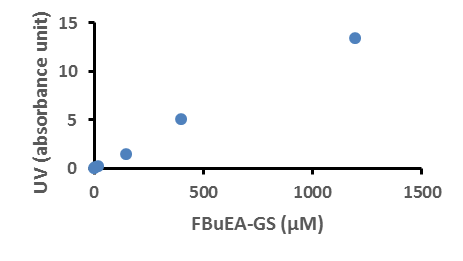

Supplement: Figure S5 — Illustration of the reponse of the UV absorption on the concentration of FBuEA-GS 3 of each HPLC chromatogram in Fig. S4. (TIF) [file pone.0104118.s005.tif]

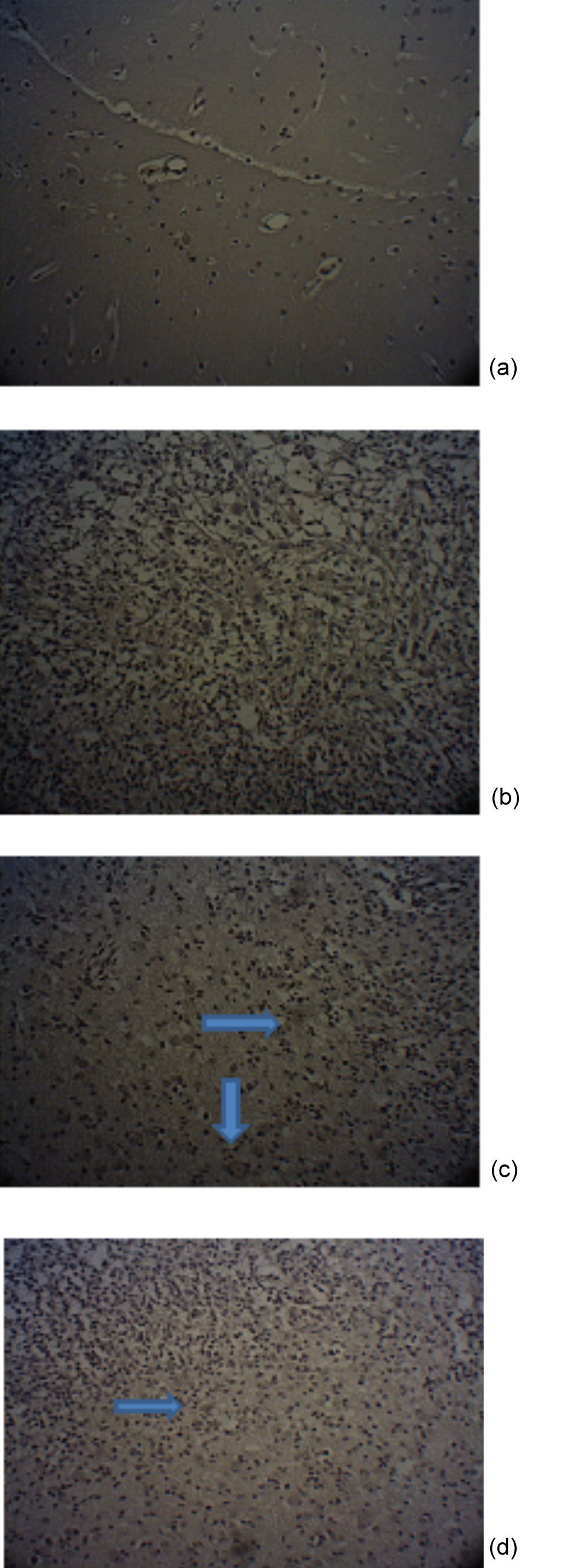

Supplement: Figure S7 — Immunohistological stainings for COXs and L-PGDS enzymes. (a) COX-1 staining of normal brain tissue, (b) COX1 staining- tumor center, (c) COX2 staining- tumor/brain margin, (d) L-PGDS staining- tumor/brain-100×. (TIF) [file pone.0104118.s007.tif]

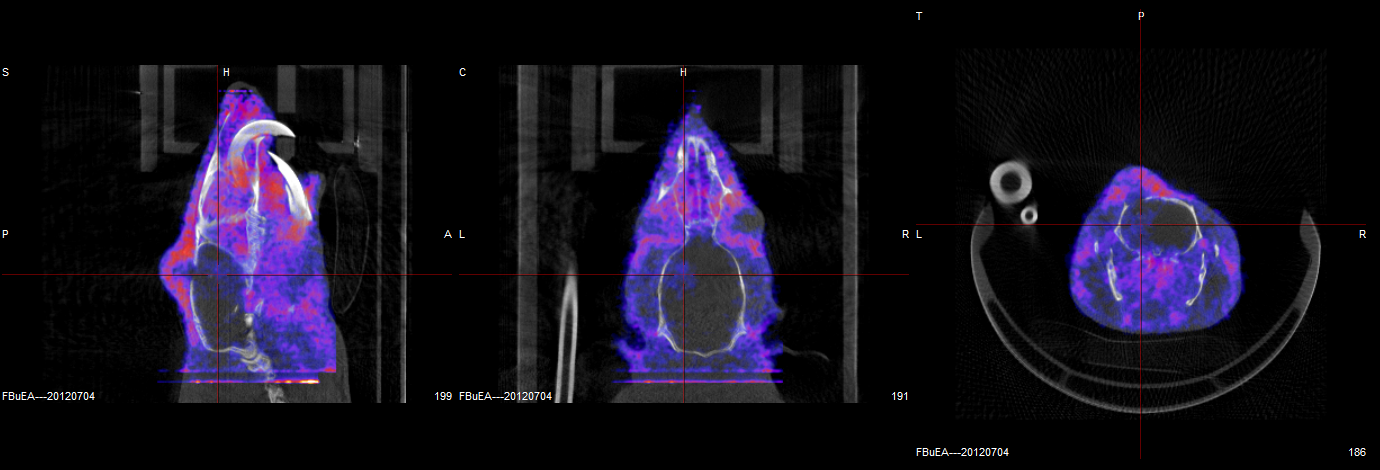

Supplement: Figure S8 — Fused CT-PET images of a C6-glioma rat for confirmation of the tumor implantation using the second PET scanner (nanoPET/CT, MEDISO Inc). From left to right: sagittal image, coronal image and transverse image. Injection dose: 1.085 mCi/0.2 mL. Images were taken from the mean of 0–60 min. (TIF) [file pone.0104118.s008.tif]
