# Supplementary material for: 18F-Glutathione Conjugate as a PET Tracer for Imaging Tumors that Overexpress L-PGDS Enzyme
Source: PLoS One. 2014 Aug 11;9(8):e104118. doi: 10.1371/journal.pone.0104118 (PMC4128654; doi:10.1371/journal.pone.0104118)
Supplement: Table S1 — Protocols for formation of PGD2. (DOCX) [file pone.0104118.s009.docx]

**Table S1.** Protocols for formation of PGD2

| **Entry** | | | | | | |
| --- | --- | --- | --- | --- | --- | --- |
| **stage** | **control** | | **initial** | **inhibitor 1**  **(uridine)** | **inhibitor 2**  **(AT-56)** | **inhibitor 3**  **(FBuEA-GS)** |
| 1 | 470 uL reaction buffer | | 460 uL reaction buffer | 450 uL reaction buffer | 450 uL reaction buffer | 450 uL reaction buffer |
| 2 | 20 uL dithiothreitol (DTT) | | | | | |
| 3 | - | | 10 uL L-PGDs | | | |
| 4 | - | | - | 10 uL uridine | 10 uL AT-56 | 10 uL FBuEA-GS |
| 5 | 10 uL PGH_2_ | | | | | |
| 6 | Shake for 1 min | | | | | |
| 6 | Add HCl (1 M, 50 uL) | | | | | |
| 7 | 10 uL FeCl_2_ | | | | | |
| 8 | Dilution (10000 fold) using EIA buffer | Dilute to 2000 fold using EIA buffer | | | | |

EIA: enzymatic immuno assay
