# Supplementary material for: 18F-Glutathione Conjugate as a PET Tracer for Imaging Tumors that Overexpress L-PGDS Enzyme
Source: PLoS One. 2014 Aug 11;9(8):e104118. doi: 10.1371/journal.pone.0104118 (PMC4128654; doi:10.1371/journal.pone.0104118)
Supplement: Table S2 — Protocols for enzymatic immunological assay. (DOCX) [file pone.0104118.s010.docx]

**Table S2.** Protocols for enzymatic immunological assay

| **Entry** | | | | | |
| --- | --- | --- | --- | --- | --- |
| **stage** | **control** | **initial** | **inhibitor 1 (uridine)** | **inhibitor 2 (FBuEA-GS)** | **inhibitor 3 (AT-56)** |
| 1 | 50 uL from part A | 50 uL from part A | 50 uL from part A | 50 uL from part A | 50 uL from part A |
| 2 | 50 uL tracer | 50 uL tracer | 50 uL tracer | 50 uL tracer | 50 uL tracer |
| 3 | 50 uL antibody | 50 uL antibody | 50 uL antibody | 50 uL antibody | 50 uL antibody |
| 4 | Sealed with parafilm and wait for 2 hr | | | | |
| 5 | Wash with buffer (150 uL) for 5 times | | | | |
| 6 | 200 uL Ellman’s reagent | | | | |
| 7 | Sealed with parafilm under dark for 1hr | | | | |
| 8 | Measure the absorbance at λ = 410 nm | | | | |

tracer: acetylcholinesterase linked PGD2, antibody: specific to recognition of PGD2, Ellman’s reagent: 5,5’-dithiobis-(2-nitrobenzoic acid)
