# Supplementary material for: 18F-Glutathione Conjugate as a PET Tracer for Imaging Tumors that Overexpress L-PGDS Enzyme
Source: PLoS One. 2014 Aug 11;9(8):e104118. doi: 10.1371/journal.pone.0104118 (PMC4128654; doi:10.1371/journal.pone.0104118)
Supplement: Table S3 — Enzymes used for radioactive ligand binding assay. (DOCX) [file pone.0104118.s011.docx]

**Table S3.** Enzymes used for radioactive ligand binding assay

| **Enzymes used** | **Stock (uints/ volume)** | **Mixing condition** | **Adjusted concentration** |
| --- | --- | --- | --- |
| PGES for both microsomal and human recombinant | 500 units/200 μL | PGES (150 μL, 375 units) and [^18^F]FBuEA-GS **3** (20 μL) | 2.2  units/μL |
| PGDS (lipocalin-type; mouse recombinant) | 250 μg / 200 μL | PGDS (150 μL, 0.4 units) and [^18^F]FBuEA-GS **3** (20 μL) | 2.4×10^-3^  units/μL |
| PGDS (lipocalin-type; rat recombinant) | 250 μg / 200 μL | PGDS (150 μL, 0.4 units) and [^18^F]FBuEA-GS **3** (20 μL) | 2.4×10^-3^  units/μL |
| PGDS (lipocalin-type; human recombinant) | 250 μg / 200 μL | PGDS (150 μL, 0.4 units) and [^18^F]FBuEA-GS **3** (20 μL) | 2.4×10^-3^  units/μL |
| COX-1 | 5000 units / 200 μL | COX-1 (100 μL, 250 units) and [^18^F]FBuEA-GS **3** (20 μL) | 20  units/μL |
| COX-2 | 5000 units / 600 μL | COX-2 (300 μL, 2500 units) and [^18^F]FBuEA-GS **3** (20 μL) | 7.8  units/μL |
| GST-α | 0.05 units/  (10 μL) | GST-α (5 μL, 0.025 units), [^18^F]FBuEA-GS **3** (10 μL) and phosphate pH = 7.3 (20 μL) | 7×10^-4^  units/μL |
| GST-π | 0.0325 units/  (5 μL) | GST-π (2.5 μL, 0.01625 units), [^18^F]FBuEA-GS **3** (10 μL) and phosphate pH = 7.3 (20 μL) | 5×10^-4^ units/μL |
