# Supplementary material for: 18F-Glutathione Conjugate as a PET Tracer for Imaging Tumors that Overexpress L-PGDS Enzyme
Source: PLoS One. 2014 Aug 11;9(8):e104118. doi: 10.1371/journal.pone.0104118 (PMC4128654; doi:10.1371/journal.pone.0104118)
Supplement: Table S4 — Tabulation for the response of the UV absorption on the concentration of FBuEA-GS 3 of each HPLC chromatogram in Fig. S2. (DOCX) [file pone.0104118.s012.docx]

**Table S4.** Tabulation for the response of the UV absorption on the concentration of FBuEA-GS **3** of each HPLC chromatogram in Fig. S2

|  | **FBuEA-GS (μM)** | | | | | | |
| --- | --- | --- | --- | --- | --- | --- | --- |
|  | 0 | 1 | 7.5 | 20 | 150 | 400 | 1200 |
| **UV integral**  **(AU)** | 0.00145 | 0.00864 | 0.07787 | 0.18391 | 1.28835 | 4.71801 | 14.8279 |

AU: arbitrary unit
