# Supplementary material for: 18F-Glutathione Conjugate as a PET Tracer for Imaging Tumors that Overexpress L-PGDS Enzyme
Source: PLoS One. 2014 Aug 11;9(8):e104118. doi: 10.1371/journal.pone.0104118 (PMC4128654; doi:10.1371/journal.pone.0104118)
Supplement: Table S5 — Tabulation for the response of the UV absorption on the concentration of FBuEA-GS 3 of each HPLC chromatogram in Fig. S4. (DOCX) [file pone.0104118.s013.docx]

**Table S5.** Tabulation for the response of the UV absorption on the concentration of FBuEA-GS **3** of each HPLC chromatogram in Fig. S4.

|  | **[^18^F]FBuEA-GS (μM)** | | | | | | |
| --- | --- | --- | --- | --- | --- | --- | --- |
|  | 0 | 1 | 7.5 | 20 | 150 | 400 | 1200 |
| **UV peak**  **(AU)** | 0.00143 | 0.00631 | 0.08263 | 0.16747 | 1.47014 | 4.99203 | 13.3452 |

AU: arbitrary unit
