# Supplementary material for: 18F-Glutathione Conjugate as a PET Tracer for Imaging Tumors that Overexpress L-PGDS Enzyme
Source: PLoS One. 2014 Aug 11;9(8):e104118. doi: 10.1371/journal.pone.0104118 (PMC4128654; doi:10.1371/journal.pone.0104118)
Supplement: Table S6 — Tabulation for the integrals of the peaks corresponding to the bounded fom and free form in the HPLC chromatogram of [18F]FBuEA-GS 3 with L-PGDS. (DOCX) [file pone.0104118.s014.docx]

**Table S6.** Tabulation for the integrals of the peaks corresponding to the bounded fom and free form in the HPLC chromatogram of [^18^F]FBuEA-GS **3** with L-PGDS.

Group A (5 sec. equilibrium)

| Concentration of FBuEA-GS **3** added (μM) | integral of the bonded form | integral of the free form | sum of the two forms |
| --- | --- | --- | --- |
| 0 | 1001.12 | 5428.64 | 6429.756725 |
| 1 | 836.8599 | 5804.340725 | 6641.20065 |
| 7.5 | 784.2733 | 5815.044725 | 6599.318 |
| 20 | 762.9938 | 5834.950925 | 6597.944675 |
| 150 | 568.2692 | 5872.867825 | 6441.137025 |
| 400 | 295.9052 | 6282.7765 | 6578.681725 |
| 1200 | 360.0933 | 5985.3802 | 6345.473475 |

Group B (10 min. equilibrium)

| Concentration of FBuEA-GS **3** added (μM) | integral of the bonded form | integral of the free form | sum of the two forms |
| --- | --- | --- | --- |
| 0 | 833.1892 | 5554.864975 | 6388.054175 |
| 1 | 844.3629 | 5650.7216 | 6495.0845 |
| 7.5 | 1016.404 | 6927.162625 | 7943.56635 |
| 20 | 834.9226 | 5915.824125 | 6750.7467 |
| 150 | 515.8714 | 6476.76715 | 6992.6385 |
| 400 | 442.4984 | 6240.6991 | 6683.197475 |
| 1200 | 334.4166 | 6281.74525 | 6616.161825 |
